# Investigation of urinary metabolomics in a phase I hookworm vaccine trial in Gabon

**DOI:** 10.1371/journal.pone.0275013

**Published:** 2022-09-26

**Authors:** Madeleine Eunice Betouke Ongwe, Yoanne D. Mouwenda, Koen A. Stam, Peter G. Kremsner, Bertrand Lell, David Diemert, Jeff Bethony, Maria E. Bottazzi, Peter J. Hotez, Remko V. Leeuwen, Martin P. Grobusch, Ayola A. Adegnika, Oleg A. Mayboroda, Maria Yazdanbakhsh

**Affiliations:** 1 Department of Parasitology, Leiden University Medical Center, Leiden, The Netherlands; 2 Centre de Recherches Médicales de Lambaréné, Lambaréné, Gabon; 3 Institut de Recherches en Écologie Tropicale, Centre National de la Recherche Scientifique et Technologique (CENAREST), Lambaréné, Gabon; 4 Institut für Tropenmedizin, Eberhad Karls Universität Tübingen, Tübingen, Germany; 5 Division of Infectious Diseases and Tropical Medicine, Department of Medicine I, Medical University of Vienna, Vienna, Austria; 6 George Washington University, Washington, DC, United States of America; 7 Texas Children’s Hospital Center for Vaccine Development, National School of Tropical Medicine, Baylor College of Medicine, Houston, TX, United States of America; 8 Amsterdam Institute for Global Health and Development, Amsterdam, The Netherlands; 9 Department of Infectious Diseases, Center of Tropical Medicine and Travel Medicine, Amsterdam University Medical Center, Amsterdam Public Health; Amsterdam Infection & Immunity, University of Amsterdam, Amsterdam, The Netherlands; 10 German Center for Infection Research (DZIF), Tübingen, Germany; 11 Center for Proteomics and Metabolomics, Leiden University Medical Center, Leiden, The Netherlands; Merck: Merck & Co Inc, UNITED STATES

## Abstract

Metabolomics provides a powerful tool to study physiological changes in response to various perturbations such as vaccination. We explored whether metabolomic changes could be seen after vaccination in a phase I trial where Gabonese adults living either in rural or semi-urban areas received the subunit hookworm vaccine candidates (*Na*-GST-1 and *Na*-APR-1 (M74) adjuvanted with Alhydrogel plus GLA-AF (n = 24) or the hepatitis B vaccine (n = 8) as control. Urine samples were collected and assayed using targeted ^1^H NMR spectroscopy. At baseline, a set of metabolites significantly distinguished rural from semi-urban individuals. The pre- and post-vaccination comparisons indicated significant changes in few metabolites but only one day after the first vaccination. There was no relationship with immunogenicity. In conclusion, in a small phase 1 trial, urinary metabolomics could distinguish volunteers with different environmental exposures and reflected the safety of the vaccines but did not show a relationship to immunogenicity.

## Introduction

Metabolomics is a post-genomic discipline which provides an analytical platform for measuring and profiling both the intermediates and the end products of metabolism; as such, it enables the study of the metabolic activity of an organism and its physiological responses to external or internal disruptions [1]. Urine, serum and plasma are the most commonly studied biofluids for exploring systemic metabolic effects such as age-related changes, dietary modulation, diurnal effects and organ toxicity [2–5].

The studies of metabolomics in vaccinology, have had the aim to identify biomarkers of adverse reactions, as well as to discover correlates or mechanisms of protection [6]. For example, a systemic response to a vaccine triggers changes in the metabolic composition of body fluids [6,7]. As vaccine immunogenicity and efficacy can be influenced by heterogeneity in environmental exposures [8–10], it would also be of interest to apply such metabolomics approaches to vaccine responses in populations living in diverse environmental conditions. Analysis of serum is an invasive method, even though blood sampling poses only a minimal risk to an individual. Yet, while taking blood samples in a vaccine trial seems a logical approach, in field studies where logistic issues and cultural reluctance to blood sampling play a role, urine sampling provides a good alternative.

Hookworm infections remain a public health concern in tropical countries. The burden of these infections is high despite large-scale deworming programs due to rapid re-infection. Therefore, an effective vaccine is needed to provide long-term protection and prevent re-infection [11]. Two enzymes, Glutathione-S-Transferase-1 (*Na*-GST-1) and Aspartic-Protease-1 (*Na*-APR-1) of the hookworm *Necator americanus*, have been identified as potential vaccine candidates [12]. These molecules are hemoglobin- and protein-degrading enzymes, components of the worm’s blood-feeding pathway and thus expected to be important for its survival. When administrated to an individual, *Na*-GST-1 and *Na*-APR-1 vaccines are intended to induce antibodies that will be ingested with the parasite’s blood meal, thereby interfering with the blood degradation and feeding process of the parasite, leading to its eventual death [13,14]. *Na*-GST-1 and *Na*-APR-1(M74) adjuvanted with Alhydrogel plus Gluco-Pyranosylphospho-Lipid an Aqueous Formulation (GLA-AF) were co-administered to healthy adult volunteers in Gabon and compared to hepatitis B vaccine in a randomized, controlled, double-blinded, dose-escalation phase I trial. Each participant received the vaccines at three time points; the safety and immunogenicity results have been published elsewhere [15].

Here we took advantage of the randomized, controlled, double-blinded, dose-escalation phase I vaccine trial assessing safety and immunogenicity of the co-administered hookworm vaccine candidates, Na-GST-1 and Na-APR-1 (M74), and the control arm, the hepatitis B vaccine, to perform a metabolomics study of samples collected before and at several time points after the vaccination. Although, the likelihood of discovering metabolites associated with intramuscular vaccination is expected to be higher in serum than in urine, value of using non-invasive methods that can also be applied to large population studies, prompted us to collect and analyze urine samples. We were encouraged by the overlapping and distinct profiles seen in several studies comparing age and sex [16,17], and asked whether urine samples will allow metabolic changes to be detected following perturbation induced by vaccination. To this end, urinary metabolite profiles were determined by a targeted ^1^H-NMR spectroscopy [18] before and after vaccination.

## Materials and methods

### Study area, sample collection and sample preparation

The trial was performed in accordance with the national ethical committee of Gabon (#0033/2014/SG/CNE) and done under an investigation new drug application (IND#016184) to the US Food and Drug administration. Written informed consent was obtained from each participant. Participants of the trial were healthy and free of HIV, hepatitis B or malaria parasites at the time of first vaccination. Any participant found to be infected with parasitic helminths was treated two weeks prior to vaccination (S1 Table). In total thirty-two Gabonese living either in semi-urban or rural areas surrounding Lambaréné (S1 Table) were enrolled in a phase I HOOKVAC trial (Clinicaltrials.gov ID: NCT02126462) and received three vaccinations (on Days 0, 28, and 180) of 30 μg (low dose group = 12) or 100 μg (high dose group = 12) of co-administered *Na*-GST-1 and *Na*-APR-1, or hepatitis B vaccine (control group = 8) as a comparator. Among them six subjects withdrew after the first immunization. Details on the clinical trial, characteristics of the study population and vaccination procedure were published elsewhere [15]. Morning urine samples were collected before (Day 0) and after each vaccination on Days 1, 3, and 7 as shown in the S1 Fig. Urine samples were first put on ice, then aliquoted and frozen at -80°C at the Centre de Recherches Médicales de Lambaréné (CERMEL) in Gabon and were transported on dry ice to Leiden for metabolomic analysis.

### NMR experiments and processing

The processing and analysis using Nuclear Magnetic Resonance (NMR) spectrometry were performed as previously reported [19]. The samples were randomised and organised in the acquisition blocks of 96 samples. Prior acquisition, urine samples were thawed and 700 μl of each urine sample was transferred to a 96 deep-well plate and centrifuged at 3000 rpm for 5 minutes. Of each sample, 630 μl of each sample was mixed with 70 μl of potassium phosphate buffer (1.5 M, pH 7.4) in 100% D_2_O containing 4 mM TSP and 2 mM NaN_3_ and transferred to a 5 mm Bruker Sample Jet NMR tubes. Subsequently the tubes were stored in the Sample Jet autosampler at 6°C while queued for measurement. ^1^H-NMR NOESY1D and JRES spectra were acquired on a Bruker 600MHz AVANCE II spectrometer equipped with a 5mm TCI cryogenic probe head and a z-gradient system using identical experimental parameters as in our earlier report. For the targeted, quantitative analysis, the raw data were exported into the Chenomx NMR suite 8.6 software (Chenomx Inc.) where 52 metabolites were annotated and quantified using a batch fit tool and manual curation. Concentrations (mM) were extracted using the known total suspended particles (TSP) concentration (0.4 mM). The quantitative data are summarized in the S1 Table.

### Data analysis

**Fig 1:**
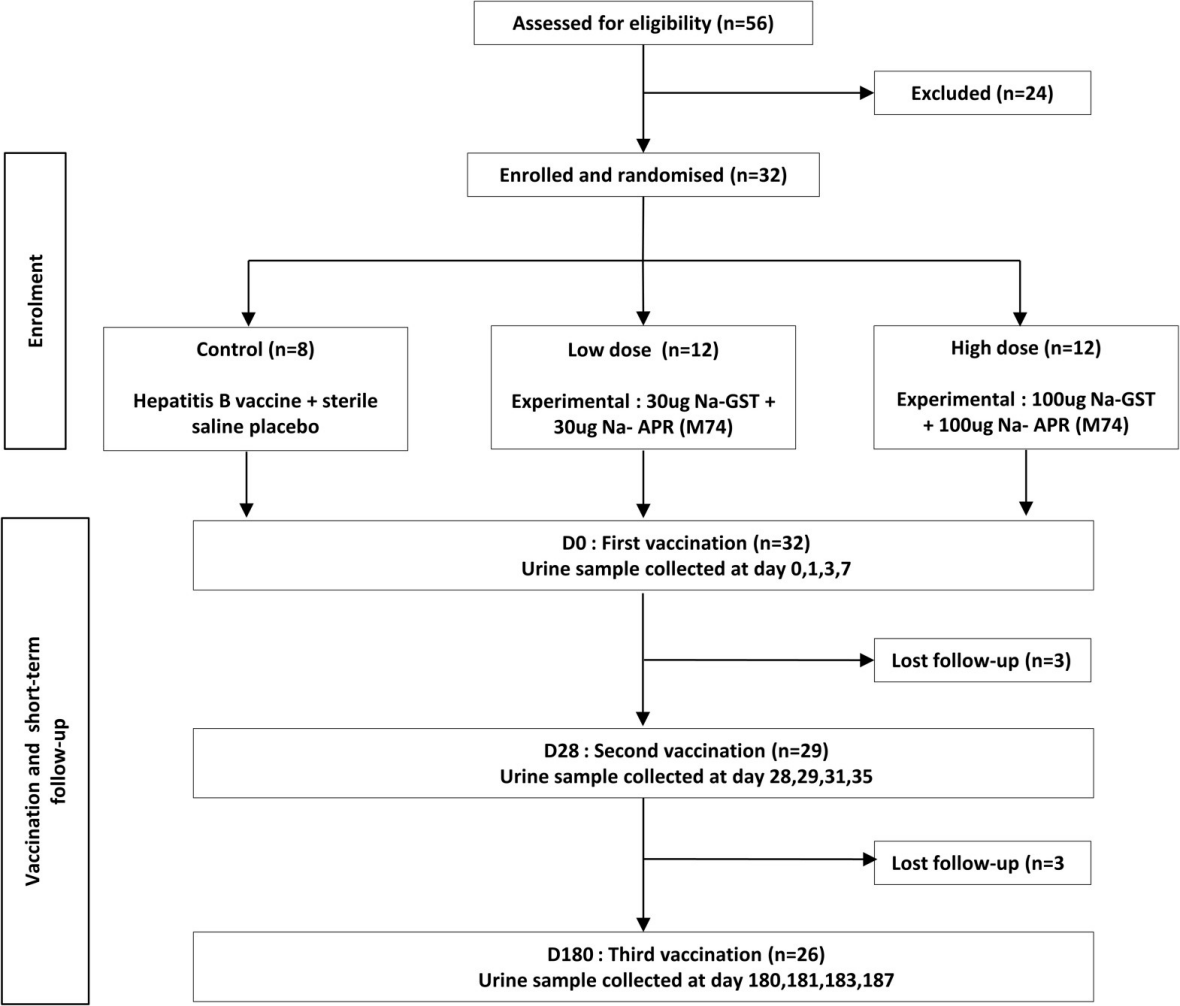
Flow chart of HOOKVAC trial. Participants were vaccinated three times at 0,1,6 months with Hepatitis B or Na-GST-1 plus Na-APR-1 (M74). Urine samples were collected before and after each vaccination at Days 0, 1, 3, 7 after each vaccination round.

All data analyses were performed using R version 4.0.2 (*www.r-project.org*). Before analysis, data were normalized using a Probabilistic quotient normalization (PQN) which compensates for possible dilution effects [20]. For baseline analysis (Day 0, prior to vaccination), exploratory analysis and multivariate modeling including Principal Component Analysis (PCA) and Partial Least Squares Discriminant analysis (PLS-DA) were used with the following packages: Rcpm 0.9.8, pcaMethods 1.78, pls 2.7–2, plsdepot 0.1.17, and caret 6.0–86. Baseline comparisons were made according to sex, whether they received anthelminthic pre-treatment (‘yes’ vs. ‘no’) for parasite infections diagnosed at screening, and geographical location of the participant’s residence (semi-urban vs rural). For post vaccination analysis, only the 26 participants who completed the study trial were considered (Fig 1). Also, due to loss of labels from half of the collected samples at the second vaccination time point, all the data related to urine samples collected at second vaccination was excluded from data analysis and only data from the first vaccination (collected on Days 0, 1, 3 and 7) and third vaccination (Days 180, 181 and 187) were considered for pre- and post-vaccination comparison. Some samples were also missing from the first and third vaccination time point due to the inability to be present at the collection time or unable to provide morning urine (S2 Table). These missing data were imputed using a Multivariate Imputations by chained equations (MICE) algorithm, which enables imputation of each incomplete variable by a separate model (R package *mice* 3.12.0) [21]. This MICE algorithm remains one of the few proven tools for multiple imputation of the data sets with tens to hundred variables. The method is an iterative procedure which specifies the multivariate imputation model on a variable-by-variable basis. The imputation was well performed for all selected time points, except for Day 183 where the number of the missing samples made the imputation not feasible. To analyze the effect of vaccination on the metabolic profiles of participants in comparison to baseline, a two-way ANOVA analysis (time and type of vaccine) on the first and third vaccination rounds was applied to every single metabolite using R *aov* (analysis of variance) function. The results of the analysis were corrected for family-wise errors. Graphs were made using the “ggplot2” package in R package.

## Results

### Exploratory analysis

**Fig 2:**
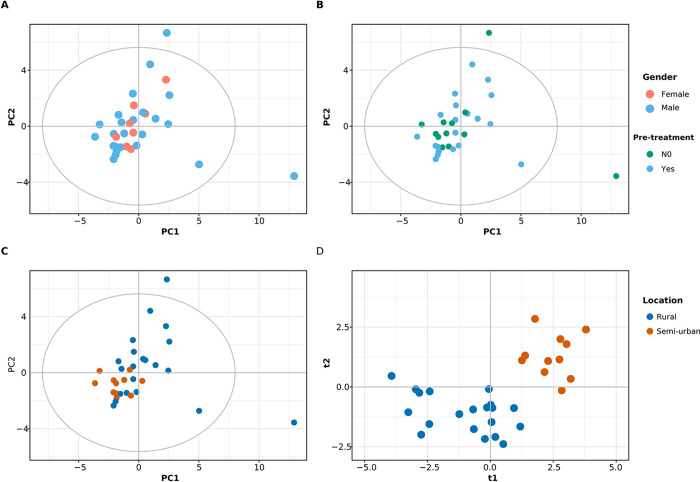
Exploratory analysis of the urine samples of volunteers at baseline. A, B and C show the score plots of PCA model according to sex (male and female), anthelmintic pre-treatment (yes or no) and area of residence (location = rural or semi-urban) respectively. PCA model built for the two first component cover 26% of the variance and 6 components were required to cover 50% of the variance. D- is a cross-validated score plot of 2 class PLS-DA model with location as class identity. Model metrics: R2X = 0.232, R2Y = 0.81, Q2 = 0.40.

**Fig 3:**
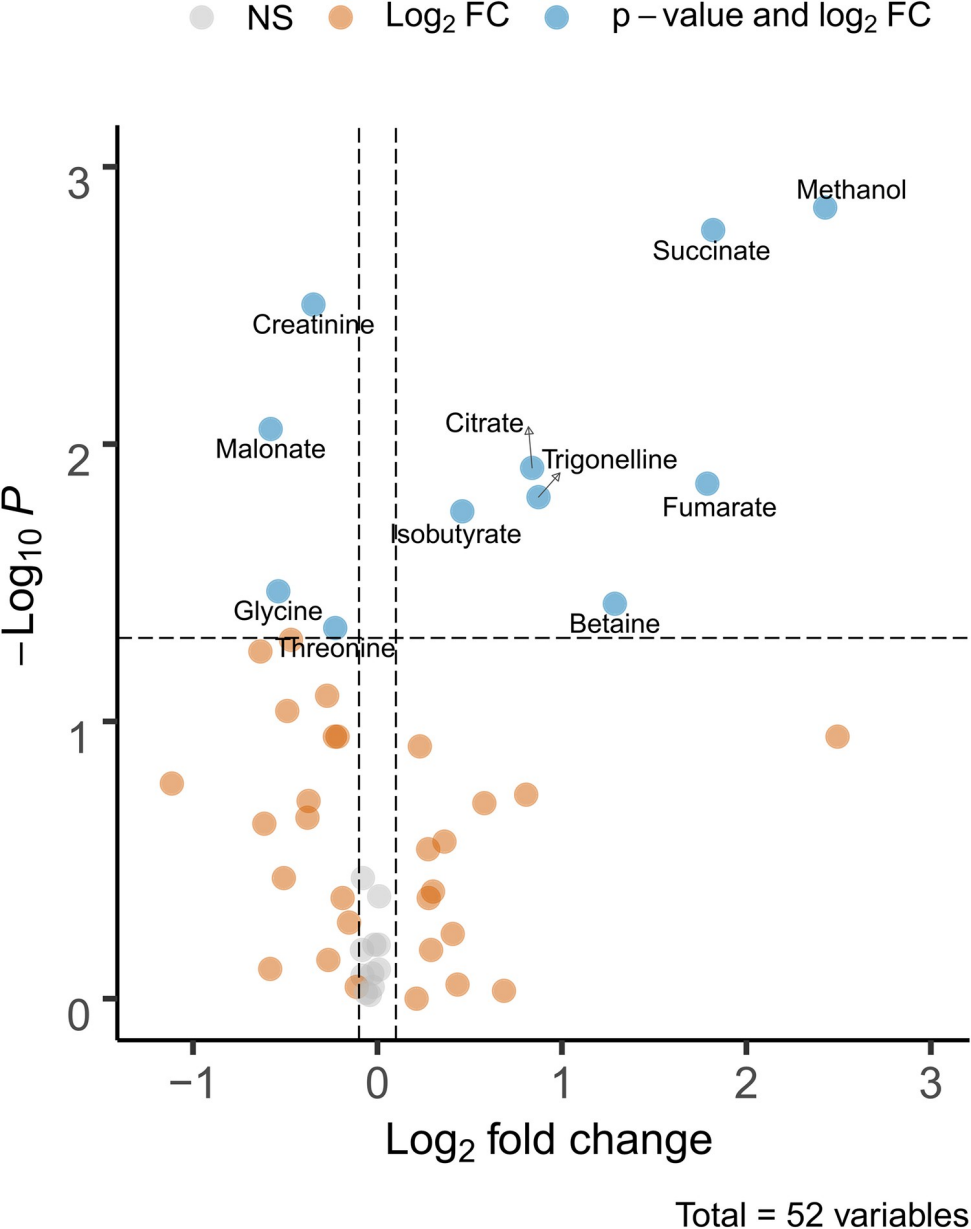
Baseline metabolites that differ between participants living in rural versus semi-urban areas. A volcano plot showing the metabolites which contribute to the differences observed between rural and semi-urban participants at baseline. NS: No change, FC: Fold change.

A principal component analysis (PCA) model built on the entire data set required 10 components to explain 50% of the variance with the first two explaining 17% of the variance. The model shows that the possible confounding factors such as gender and anthelmintic pretreatment (treatment was given prior to start of the study to ensure subjects to be free of helminth infection, n = 20) have no effect on the data (S1 Fig). At the same time, neither the time points at pre and post vaccination (S1A Fig) nor type of a vaccine and dose (S1B Fig) showed any influence on the first two principal components. However, a clustering tendency was seen according to the participant area of residence: rural or semi-urban (S1C Fig). Next, we examined whether the segregation according to the area of residence is observed before vaccination (at baseline). A PCA model built using the baseline samples required six components to explain 50% of the variance, with the first two components explaining 26% of the variance. Baseline exploratory analysis showed no influence of gender (Fig 2A) and anthelmintic pre-treatment (Fig 2B) and a tendency to clustering according to area of residence (Fig 2C) as previously observed in S1 Fig. Thus, to investigate further the difference between the subjects in rural and semi-urban location, we used a multivariate regression model, namely the Partial Least-Squares Discriminant Analysis–PLS-DA. The score plot of the two class PLS-DA model using rural or semi-urban location as the class identity is shown in Fig 2D. This plot shows a clear clustering according to geographical location. The model metrics—R2X = 0.232, R2Y = 0.81, Q2 = 0.40 indicate that while the model explains more than 80% class-related variance (R2Y), its predictive ability (Q) remains only 40%. The limited predictive ability of the regression model indicates a weak model and suggests a possibility of a limited number of metabolites differing between rural and semi-urban participants. We decided to support the results of the multivariate model with a univariate test. The results of the univariate test are summarised in a volcano plot (Fig 3): the urinary concentrations of citrate, isobutyrate, betaine, fumarate, succinate, trigonelline and methanol are significantly higher in participants living in rural areas; the participants from semi-urban areas had higher concentrations of creatinine, malonate, glycine and threonine.

### Metabolic response to the vaccination

**Fig 4:**
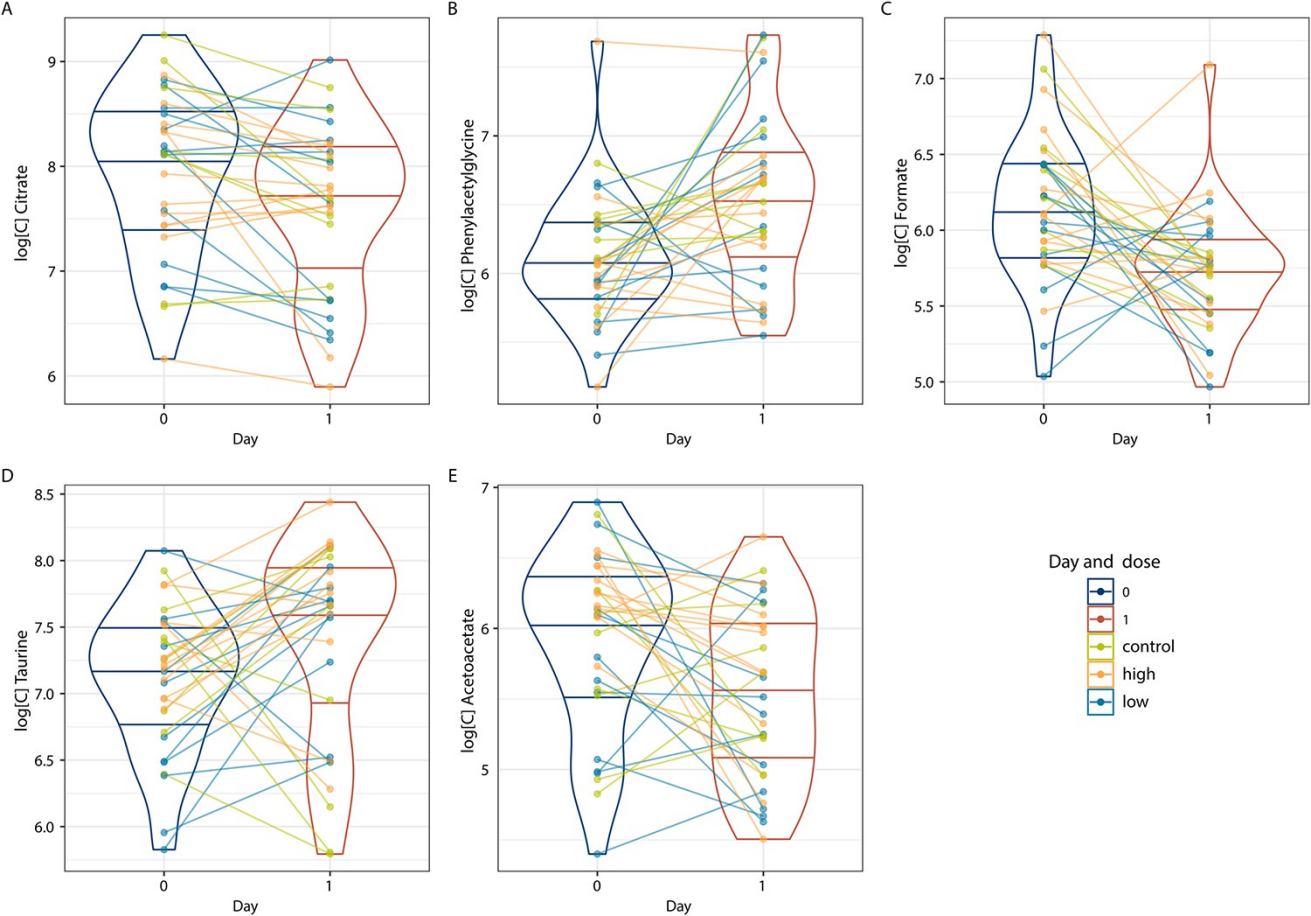
Metabolites that changed at day 1 post vaccination. A, B, C, D and E show plots of metabolites that changed significantly at day 1 post vaccination. Y axis corresponds to metabolite log transformed concentration and X axis to timepoint. Dot and line are colored according to vaccine type (control = Hepatitis B, low = low dose HOOKVAC and high = high dose of HOOKVAC). Horizontal lines indicate 0.25, 0.50, 0.75 quantiles. HOOKVAC: Hookworm vaccine candidates comprising of Na-GST-1 and Na-APR-1 (M74).

To get insight into the possible effects of the vaccination on the urinary metabolome we performed an exploratory multivariate analysis on the data of the first and third rounds of vaccination separately. The S2 Fig shows that neither first (S2A and S2B Fig), nor third (S2C and S2D Fig) round of vaccination trigger strong changes in the urinary metabolome. Next, we tried to reveal the vaccination related changes using the multivariate regression models. However, the resulting model was weak, and its discriminatory power remained on a level of random class assignment. To rule out a possibility that multivariate analysis “misses” potential vaccine related changes we performed a two-way ANOVA analysis (time and type of vaccine). We build an individual ANOVA model for every metabolite using the first two time point of each vaccination round (Day 0—Day 1 for the first round and Day180 –Day 181 for the third round). The results showed no statistically significant changes related to the type of vaccine administered. Only few metabolites, namely citrate, phenylacetylglycine, taurine, formate and acetoacetate change significantly between the baseline and Day 1 (Fig 4). No significant differences were detected between Day 180 and Day 181.

## Discussion

Here we report for the first time 1H-NMR metabolomics analysis of urine of the subjects participating in a clinical trial testing a hookworm vaccine candidate (HOOKVAC comprising of Na-GST-1 and Na-APR-1 (M74)). The experimental HOOKVAC and the hepatitis B vaccine were administered to individuals living in Gabon, a country with high prevalence of helminth infections [22,23]. We found that at baseline area of residence of participants (rural or semi-urban) influences a set of the urinary metabolites (Fig 3). Association between lifestyle, dietary habits, microbiota composition and metabolic profiles of the body fluids has been reported before [24,25].

The principal finding of our report is that administration of this experimental HOOKVAC nor that of Hepatitis B vaccine affects metabolic composition of urine and do not trigger any metabolic stress response. This finding is in agreement with previously reported assessment of the vaccine safety [15] showing no severe adverse effects which usually trigger strong changes in the urinary metabolome [26]. However, we observed some significant changes in citrate, phenylacetylglycine, formate, taurine and acetoacetate at Day 1 post vaccination. This is in agreement with a study conducted in a herpes zoster vaccine trial where metabolite changes were most pronounced on Day 1 after vaccination [7]. Regarding the metabolites identified, phenylacetylglycine levels have been seen to increase in animal models in widely different conditions, such as hypertension [27] or chronic schistosomiasis [28], and can be derived from microbial metabolism, but a rapid increase, one day after vaccination, as seen here, has not been reported before. An increase in urinary taurine is often associated with hepatotoxicity [29]. Yet, since taurine is a semi-essential beta amino acid, its fluctuations could also be linked to the dietary habits [30]. It is possible that the changes observed one day after the first vaccination were driven by the effect of the vaccine adjuvants, which was Alhydrogel for all vaccines and additional GLA-AF for the hookworm vaccine candidates. The lack of a change in the same metabolites after the third vaccination, might suggest a degree of desensitization to adjuvant effect.

To summarize, there is a growing interest in metabolic signatures of responses to infections and vaccines [26]. It has been recently shown in a study of North American subjects vaccinated with herpes zoster vaccine that the changes of plasma metabolites can predict immunogenicity or/and protection [7]. The study was conducted in a larger number of subjects (n = 77) and used a live-attenuated vaccine. Such vaccines are generally considered to be more potent inducers of immune responses compared with subunit vaccines [31]. In the current report we do not find a strong effect of the subunit vaccines, HOOKVAC or hepatitis B vaccine on urinary metabolite concentrations of the study participants indicating that for subunit vaccines, there is no potential for sampling of urine to identify biomarkers for vaccine immunogenicity or efficacy. The question remains whether sampling of urine would show changes in metabolites following administration of live attenuated vaccines, which might be more potent in inducing a response [7]. However, to link metabolic changes to immunogenicity or mechanisms of action of the subunit vaccines a study on the plasma/serum samples would be needed.

## Supporting information

S1 Checklist (DOC)

S1 Fig An exploratory analysis of the entire dataset. A, B C, D and E show the score plots of PCA model colored according to time of the sampling, type of vaccine used, area of residence (location = rural or semi-urban), gender (male and female) and anthelmintic pre-treatment (yes or no) respectively. PCA model built for the two first component cover 17% of the variance and 10 components was required to cover 50% of the variance.
(TIF)

S2 Fig An exploratory analysis of metabolic response to vaccination on the first (A, B) and the third (C, D) vaccination rounds. The score plots of the PCA models are colored according to time of the sampling (A, C) type of vaccine used (B, D). 17% of the variance is covered by the first 2 components and 8 components are needed to explain 50% of the variance.
(TIF)

S1 Table. Data of quantified metabolites (XLSX)

S2 Table Missing urine samples during HOOKVAC trial. Overview of missing data in blue. Data from Days 28, 29, 31 and 35 corresponding to second vaccination were excluded for data analysis.
(XLSX)
