# Supplementary material for: Investigation of urinary metabolomics in a phase I hookworm vaccine trial in Gabon
Source: PLoS One. 2022 Sep 26;17(9):e0275013. doi: 10.1371/journal.pone.0275013 (PMC9512193; doi:10.1371/journal.pone.0275013)
Supplement: S1 Checklist — (DOC) [file pone.0275013.s001.doc]

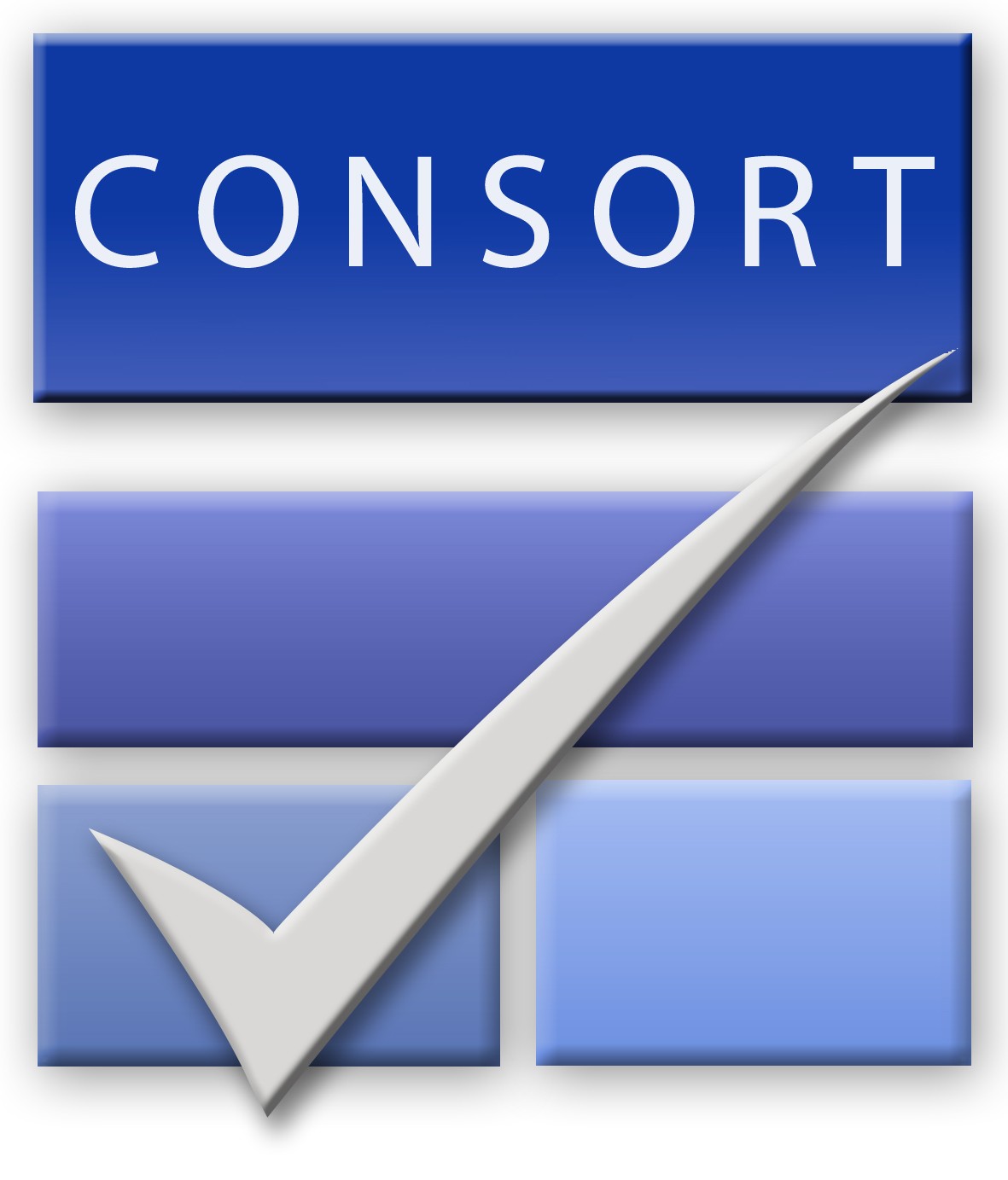
CONSORT 2010 checklist of information to include when reporting a randomised trial*

| Section/Topic | Item No | Checklist item | Reported on page No |
| --- | --- | --- | --- |
| Title and abstract | | | |
|  | 1a | Identification as a randomised trial in the title | Protocol Page 9 |
|  | 1b | Structured summary of trial design, methods, results, and conclusions (for specific guidance see CONSORT for abstracts) | Protocol Page 9-11 |
| Introduction | | | |
| Background and objectives | 2a | Scientific background and explanation of rationale | Protocol Page 12-13 |
|  | 2b | Specific objectives or hypotheses | Protocol Page 24 |
| Methods | | | |
| Trial design | 3a | Description of trial design (such as parallel, factorial) including allocation ratio | Protocol Page 25-26 |
|  | 3b | Important changes to methods after trial commencement (such as eligibility criteria), with reasons | Protocol Page 28 |
| Participants | 4a | Eligibility criteria for participants | Protocol Page 29 |
|  | 4b | Settings and locations where the data were collected | Protocol Page 49-50 |
| Interventions | 5 | The interventions for each group with sufficient details to allow replication, including how and when they were actually administered | Protocol Page 32-35 |
| Outcomes | 6a | Completely defined pre-specified primary and secondary outcome measures, including how and when they were assessed | Protocol Page 51-53 |
|  | 6b | Any changes to trial outcomes after the trial commenced, with reasons | Protocol Page 56-57 |
| Sample size | 7a | How sample size was determined | Protocol Page 54 |
|  | 7b | When applicable, explanation of any interim analyses and stopping guidelines | NA |
| Randomisation: |  |  |  |
| Sequence generation | 8a | Method used to generate the random allocation sequence | Protocol Page 34 |
|  | 8b | Type of randomisation; details of any restriction (such as blocking and block size) | Protocol Page 34 |
| Allocation concealment mechanism | 9 | Mechanism used to implement the random allocation sequence (such as sequentially numbered containers), describing any steps taken to conceal the sequence until interventions were assigned | Protocol Page 34 |
| Implementation | 10 | Who generated the random allocation sequence, who enrolled participants, and who assigned participants to interventions | Protocol Page 34 |
| Blinding | 11a | If done, who was blinded after assignment to interventions (for example, participants, care providers, those assessing outcomes) and how | Protocol Page 34 |
|  | 11b | If relevant, description of the similarity of interventions | NA |
| Statistical methods | 12a | Statistical methods used to compare groups for primary and secondary outcomes | Protocol Page 51-52 |
|  | 12b | Methods for additional analyses, such as subgroup analyses and adjusted analyses | Protocol Page 52-53 |
| Results | | | |
| Participant flow (a diagram is strongly recommended) | 13a | For each group, the numbers of participants who were randomly assigned, received intended treatment, and were analysed for the primary outcome | Manuscript Page 4 lign 97-98 |
|  | 13b | For each group, losses and exclusions after randomisation, together with reasons | Manuscript Page 4 lign 97-98 |
| Recruitment | 14a | Dates defining the periods of recruitment and follow-up | Manuscript Page 4 lign 97-98 |
|  | 14b | Why the trial ended or was stopped | NA |
| Baseline data | 15 | A table showing baseline demographic and clinical characteristics for each group | Manuscript Page 4 lign 97-98 |
| Numbers analysed | 16 | For each group, number of participants (denominator) included in each analysis and whether the analysis was by original assigned groups | Manuscript Page 4 lign 97-98 |
| Outcomes and estimation | 17a | For each primary and secondary outcome, results for each group, and the estimated effect size and its precision (such as 95% confidence interval) | Manuscript Page 4 lign 97-98 |
|  | 17b | For binary outcomes, presentation of both absolute and relative effect sizes is recommended | Manuscript Page 4 lign 97-98 |
| Ancillary analyses | 18 | Results of any other analyses performed, including subgroup analyses and adjusted analyses, distinguishing pre-specified from exploratory | Manuscript Page 4 lign 97-98 |
| Harms | 19 | All important harms or unintended effects in each group (for specific guidance see CONSORT for harms) | NA |
| Discussion | | | |
| Limitations | 20 | Trial limitations, addressing sources of potential bias, imprecision, and, if relevant, multiplicity of analyses | Manuscript Page 4 lign 97-98 |
| Generalisability | 21 | Generalisability (external validity, applicability) of the trial findings | Manuscript Page 4 lign 97-98 |
| Interpretation | 22 | Interpretation consistent with results, balancing benefits and harms, and considering other relevant evidence | Manuscript Page 4 lign 97-98 |
| Other information | | |  |
| Registration | 23 | Registration number and name of trial registry | Manuscript Page 4 lign 97-98 |
| Protocol | 24 | Where the full trial protocol can be accessed, if available | supplement |
| Funding | 25 | Sources of funding and other support (such as supply of drugs), role of funders | Manuscript Page 4 lign 97-98 |

*We strongly recommend reading this statement in conjunction with the CONSORT 2010 Explanation and Elaboration for important clarifications on all the items. If relevant, we also recommend reading CONSORT extensions for cluster randomised trials, non-inferiority and equivalence trials, non-pharmacological treatments, herbal interventions, and pragmatic trials.

Additional extensions are forthcoming: for those and for up to date references relevant to this checklist, see [www.consort-statement.org](http://www.consort-statement.org/).
