# Supplementary figures and images for: Investigation of urinary metabolomics in a phase I hookworm vaccine trial in Gabon
Source: PLoS One. 2022 Sep 26;17(9):e0275013. doi: 10.1371/journal.pone.0275013 (PMC9512193; doi:10.1371/journal.pone.0275013)

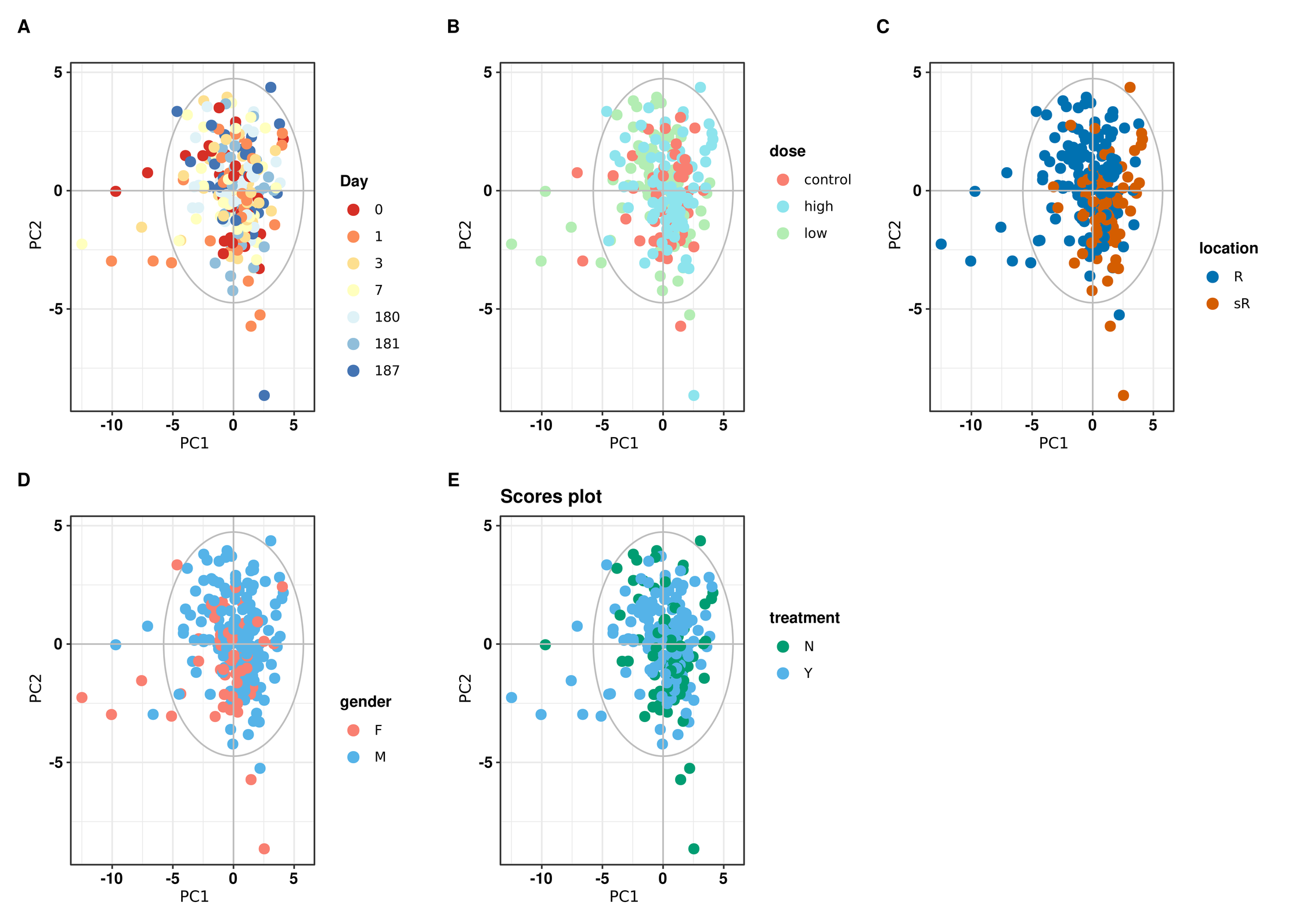

Supplement: S1 Fig — A, B C, D and E show the score plots of PCA model colored according to time of the sampling, type of vaccine used, area of residence (location = rural or semi-urban), gender (male and female) and anthelmintic pre-treatment (yes or no) respectively. PCA model built for the two first component cover 17% of the variance and 10 components was required to cover 50% of the variance. (TIF) [file pone.0275013.s002.tif]

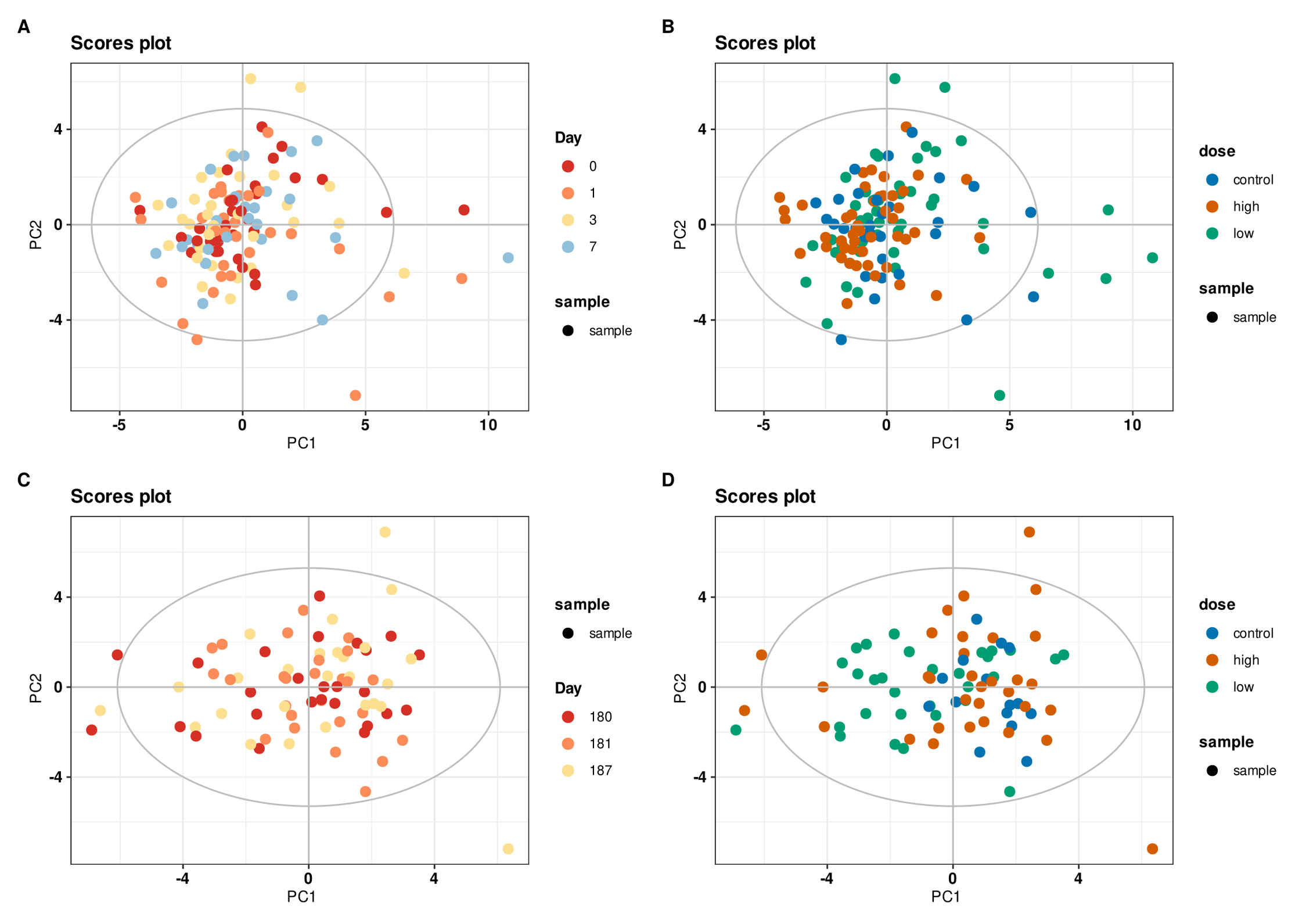

Supplement: S2 Fig — The score plots of the PCA models are colored according to time of the sampling (A, C) type of vaccine used (B, D). 17% of the variance is covered by the first 2 components and 8 components are needed to explain 50% of the variance. (TIF) [file pone.0275013.s003.tif]
